# I-Gel is a suitable alternative to endotracheal tubes in the laparoscopic pneumoperitoneum and trendelenburg position

**DOI:** 10.1186/s12871-016-0291-1

**Published:** 2017-01-06

**Authors:** Chih-Jun Lai, Chih-Min Liu, Chun-Yu Wu, Feng-Fang Tsai, Ping-Huei Tseng, Shou-Zen Fan

**Affiliations:** 1 Department of Anesthesiology, National Taiwan University Hospital Hsin-Chu Branch, NO. 25, Lane 442, Sec.1, Jingguo Rd., Hsinchu City, 30059 Taiwan (R.O.C.); 2 Department of Anesthesiology, National Taiwan University Hospital, No. 7, Zhung Shan S. Rd., Zhongzheng Dist., Taipei City, 10002 Taiwan (R.O.C.); 3 Department of Internal Medicine, National Taiwan University Hospital, No. 7, Zhung Shan S. Rd., Zhongzheng Dist., Taipei City, 10002 Taiwan (R.O.C.)

**Keywords:** Endotracheal tube, i-gel, Laparoscopic surgery, Leak fraction, Pneumoperitoneum, Respiratory parameters, Trendelenburg position

## Abstract

**Background:**

The use of supraglottic airway devices (SADs) in surgeries with laparoscopic pneumoperitoneum and Trendelenburg (LPT) positioning is controversial due to concerns about insufficient pulmonary ventilation and aspiration. In this prospective, randomized-controlled trial, we evaluated whether the i-gel, a new second generation SAD, provides an effective alternative to an endotracheal tube (ETT) by comparing respiratory parameters and perioperative respiratory complications in non-obese patients.

**Methods:**

In a randomized controlled trial, forty anesthetized patients with ASA I-II were divided into equally sized i-gel and ETT groups. We evaluated the respiratory parameters in the supine and LPT position in comparison between the two groups. The leak fraction was our primary outcome, which was defined as the leak volume divided by the inspired tidal volume. The leak volume was the difference between the inspired and expired tidal volumes. We also monitored pulmonary aspiration and respiratory complications during the perioperative period.

**Results:**

In the LPT position, there were no differences in the leak fraction (median [IQR]) between the i-gel and ETT groups (6.20[3.49] vs 6.38[3.71] %, *P* = 0.883). In the i-gel group, notably less leakage was observed in the LPT position than in the supine position (median [IQR]: 7.01[3.73] %). This phenomenon was not observed in the ETT group. The rate of postoperative sore throat was also significantly lower in the i-gel group than in the ETT group (3/17 vs 9/11). No vomitus nor any signs associated with aspiration were noted in our patients after extubation in the follow-up prior to discharge.

**Conclusions:**

The i-gel provides a suitable alternative to an ETT for surgeries with LPT positioning in non-obese patients.

**Trial registration:**

Registered at Clinicaltrials.gov NCT02462915, registered on 1 June 2015.

**Electronic supplementary material:**

The online version of this article (doi:10.1186/s12871-016-0291-1) contains supplementary material, which is available to authorized users.

## Background

Surgeries that require laparoscopic pneumoperitoneum and Trendelenburg (LPT) positioning are becoming popular. However, this positioning causes a cephalic shifting of viscera and diaphragm. The changes in respiratory mechanics following patient receiving LPT position, may result in increased airway pressure [1]. It has also been associated with potential increases in episodes of gastric regurgitation [2, 3]. Tracheal intubation to secure airway is the gold standard in surgeries requiring LPT positioning. Recently, trends in airway management have progressed from using an endotracheal tube (ETT) to a supraglottic airway device (SAD) because of the advantages that such devices confer [4–6]. However, the use of SADs in surgeries requiring LPT positioning remains controversial because of the increased risk of insufficient ventilation and pulmonary aspiration [7–9].

The second-generation SADs with gastric channel provide higher sealing pressures and more complete airway protection than the laryngeal mask airway classic [10–13]. The i-gel (Intersurgical, Wokingham, UK) is a new second-generation SAD. It includes the non-inflatable cuff, and a buccal stabilizer to prevent malposition [14]. It provides lower respiratory complications and is capable of sealing higher oropharyngeal leak pressures than earlier SADs [15]. The device is fabricated from styrene ethylene butadiene styrene (SEBS) [16], and provides improved sealing pressure when warming up to body temperature [17]. Although the i-gel has proven effective and safe for use in elective surgeries using positive ventilation, there is limited evidence to support the use of an i-gel in surgeries requiring LPT positioning.

In this study, we predicted that i-gel would perform comparably compared with an ETT in the LPT position by comparing respiratory parameters and perioperative complications in non-obese patients.

## Methods

### Patients and protocol

A single-center randomized controlled trial study was conducted in the period between June 2015 and December 2015. We obtained the approval from the Ethics Committee of National Taiwan University hospital (No. 201502043RINC), and registered the Clinical trial.gov (NCT02462915). Patients were included if they (1) had an American Society of Anesthesiologists physical status score I or II, (2) were aged 20–80 years old, (3) had an entire surgery time of less than 3 h, and (4) had undergone an elective gynecologic laparoscopy. Patients were excluded if they exhibited lung, heart, or brain disease; pathology of the neck or upper respiratory tract; difficult intubation; increased risk of aspiration (gastroesophageal reflux or full stomach); obesity (body mass index > 30) or pregnancy. After written the informed consent, the patient candidate randomized by a computer-generated random number table. Using the random number table, Patients were randomly allocated into two groups: 20 patients were treated using the i-gel and 20 patients were treated using ETTs. The surgeon were blind to our airway management and a blind observer recorded our outcome.

The patients in both groups received standardized anesthesia using the following procedure. Before anesthetic induction, the anesthetic machine and circuits were checked as the manufacturers’ guidelines. We attached standard monitors to our patients, such as pulse oximeter, electrocardiography, noninvasive blood pressure. Then patients received preoxygenation for 3 min without bag-and-mask ventilation to prevent stomach fullness. Induction was then performed using routine medication: fentanyl 1–2ug kg^−1^, Propofol 2–3 mg kg^−1^ and cisatracurium 0.2 mg kg^−1^. Neuromuscular blockade was then confirmed by monitoring the train-of-four stimulation until the count achieved zero (TOF = 0). Accordingly, either an ETT or an i-gel was inserted. For the i-gel group, the i-gel size selection depended on the patient’s body weight. Size three was used for patients who weighed less than 50 kg, size four for 50–90 kg, and size five for those who weighed more than 90 kg. Tracheal tube size was 7.0 for female patients. Anesthesia maintenance was obtained by end-tidal concentration of sevoflurane 2–3% in a 50% oxygen and 50% air mixture.

After the i-gel or ETT insertion, patients were ventilated using volume controlled ventilation (8 mL kg^−1^) of body weight with a respiratory rate of 8–16 breaths min^−1^, an inspiratory-to-expiratory ratio of 1:2, and 40% inspired oxygen in air with a fresh flow gas rate of 3 L min^−1^ [18]. Sufficient ventilation was defined as a square-wave with EtCO_2_ values of 30–45 cm H_2_O. If some emergencies were noted in the i-gel group, such as airway obstruction or hemodynamic unstable, the i-gel was removed. Then, the patient was recorded failure, and underwent endotracheal intubation.

**Fig. 1:**
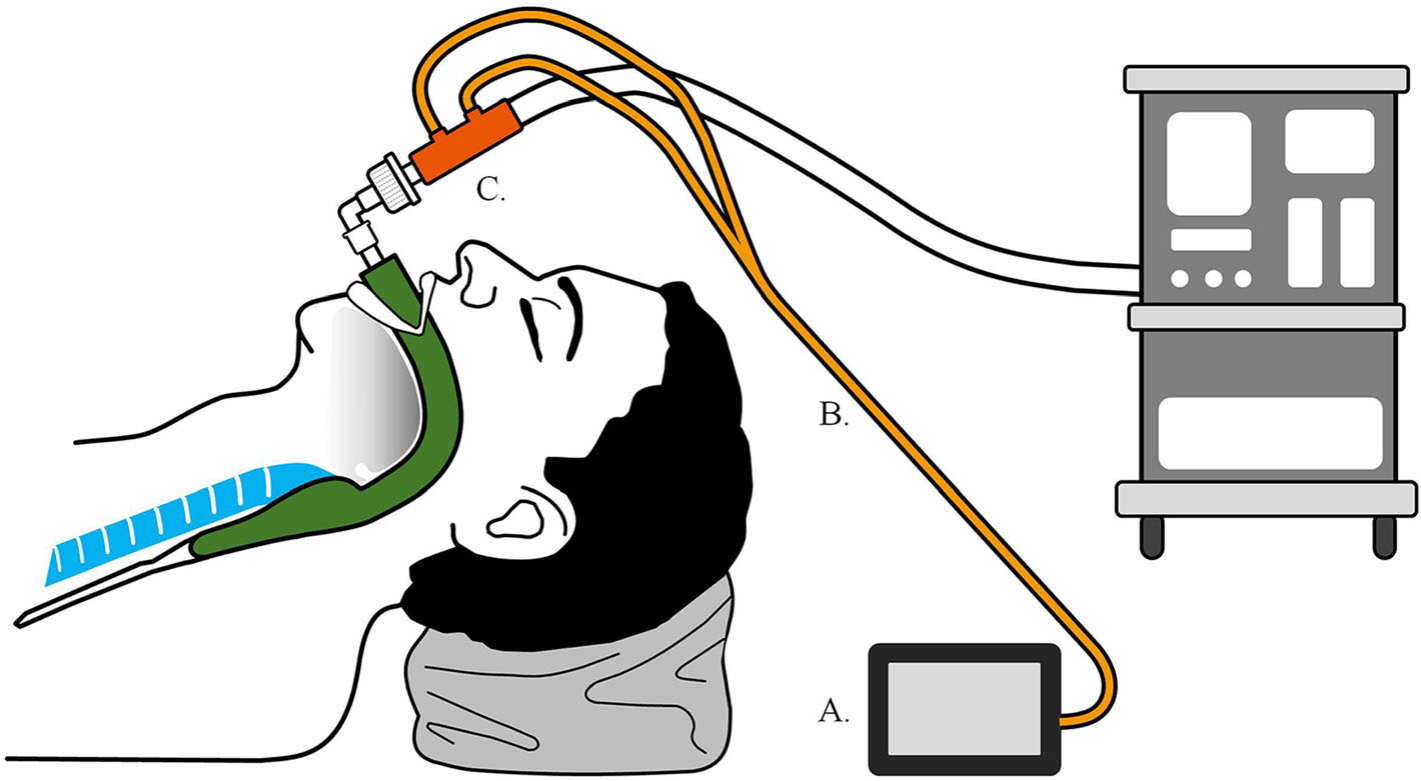
Illustration of experimental settings. *a* GE S/5 compact anesthesia monitor (GE Healthcare, Helsinki, Finland). *b* spirometry tube (GE Healthcare, Helsinki, Finland). *c* D-lite sensor (GE Healthcare, Helsinki, Finland)

To collect our data, we recorded respiratory parameters when patients were in the supine phase and LPT position, which is at 25° (AMSCO 3085 SP Surgical Table) and at which the intra-abdominal pressure is 12 mmHg. The mean values for 5 min were obtained using a GE S/5 Compact Anesthesia Monitor (GE Healthcare, Helsinki, Finland) with a spirometry tube (GE Healthcare, Helsinki, Finland) and D-lite sensor (GE Healthcare, Helsinki, Finland) (Fig. 1).

In our study, the primary outcome was the leak fraction. The leak fraction was subsequently defined as leak volume divided by inspired tidal volume (leak volume ∕ ITV). Leak volume was defined as the difference between the inspired tidal volume (ITV) and the expired tidal volume (ETV).

Oropharyngeal leak pressure (OLP) was recorded in the supine and LPT positions using the auscultation and plateau methods [19]. The auscultation method involved placing a stethoscope at the laryngeal inlet. We squeezed the reservoir bag, until we reached the lowest pressure at which gas could be heard escaping though the stethoscope. The plateau method involved closing the pressure-limiting valve of the circuit, and adjusting the fresh gas flow to 3 L min^−1^. We then recorded the plateau airway pressure. To avoid the possibility of gastric insufflation, the airway pressure was not allowed to exceed 30 cm H_2_O for either method. In addition, the cuff pressure of the ETT was set to 30 cmH_2_O using a pocket cuff pressure gauge [20, 21].

We suctioned the patients in the i-gel group through the gastric channel prior to extubation. Following extubation or i-gel removal we observed whether there was any vomitus in any of the patients’ mouths.

In the postoperative care unit, patients were followed-up for perioperative respiratory complications and clinical signs of aspiration. They were also observed by nurse anesthetists who were blind to the purposes of the study and had 10 or more years’ of experience for complications following the procedure. The side effects they looked for included laryngospasm, reintubation and a sore throat [22].

### Statistical analysis

In this study, the primary comparison parameter was leak fraction. To estimate the sample size, based on previous research we calculated that a difference of more than 0.2 in the leak fraction between the i-gel and the ETT would be needed to be considered a significant difference [23]. There was no existing general literature on which we could base our comparison between the i-gel and the ETT in the LPT position. We also set a standard deviation value of 0.15 based on research performed by Devitt et.al [24]. We used a two-sample study design to compare group means. Analyses using MINITAB 14 Statistical Software (Minitab Inc., State College, USA) found that 10 patients were required in each group in order for our study to have 80% power and a significance level of 5%.

Mann–Whitney Test were used to compare the respiratory parameter differences between i-gel and ETT groups. Wilcoxon Signed Ranks Test were used to compare the respiratory parameter difference within i-gel and ETT groups. Student’s t-tests and Chi-Squared tests were used to compare the continuous and categorical data, respectively. A two-sided *P* value of <0.05 was considered statistically significant. Statistical analyses were performed using SPSS 17.0 statistical software (SPSS Inc., Chicago, IL, USA).

## Results

**Fig. 2:**
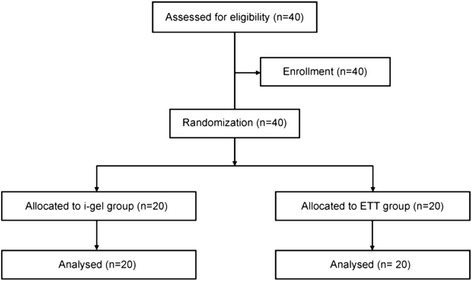
Study flow diagram. ETT: endotracheal tube

Twenty patients were enrolled into each of the i-gel and ETT groups successfully (Fig. 2). There were no significant differences in demographic data between the two groups (Table 1). All values of the measurements (the raw data) were shown in the Additional file 1 and 2.

**Table 1:** Patient characteristics

		i-gel	ETT	*P* value
		(*n* = 20)	(*n* = 20)	
Age (yr)		36.60 ± 12.03	40.65 ± 7.26	0.205
BMI		21.46 ± 3.13	21.27 ± 2.70	0.839
Surgery time (min)		111.55 ± 43.80	117.45 ± 47.07	0.684
ASA	(I)	10 (50%)	13 (65%)	0.337
	(II)	10 (50%)	7 (35%)	

*ETT* endotracheal tube

Data are means ± SD

For both the i-gel and ETT groups, peak and mean airway pressure and resistance were significantly higher in the LPT position than in the supine position (*p* < 0.001). Moreover, compliance was significantly lower in the LPT position than in the supine position (*p* < 0.001) (Table 2).

**Table 2:** Respiratory parameter changes in supine and pneumoperitoneum and Trendelenburg position

	Supine			Pneumoperitoneum and Trendelenburg		
	i-gel	ETT	*p*-value^a^	i-gel	ETT	*p*-value^b^
Leak Fraction (%)	7.01 [3.73]	5.76 [2.67]	0.341	6.20 [3.49]	6.38 [3.71]	0.883
Leak Volume (ml)	31.99 [14.54]	26.06 [9.62]	0.327	27.66 [1.73]	28.35 [18.18]	0.968
Resistance (cmH_2_O/l/s)	5.82 [1.18]	10.25 [1.03]	<0.01	7.95 [1.57]	13.13 [1.71]	<0.01
Peak Airway Pressure (cmH_2_O)	11.44 [1.43]	12.53 [1.68]	0.03	20.75 [2.75]	21.60 [3.24]	0.461
Mean Airway Pressure (cmH_2_O)	4.67 [0.71]	4.95 [0.46]	0.049	6.73 [1.33]	6.65 [0.98]	0.841
Compliance (ml/cmH_2_O)	48.45 [7.98]	46.34 [8.13]	0.62	23.02 [4.12]	23.19 [4.64]	0.779

*ETT* endotracheal tube

Data are median [IQR]

^a^ *p*-value in comparison with i-gel and ETT in supine

^b^ *p*-value in comparison with i-gel and ETT in Pneumoperitoneum and Trendelenburg position

In the LPT position, the median [IQR] leak fractions for the i-gel and ETT groups were 6.20 [3.49] % and 6.38 [3.71] %, respectively (*P* = 0.883). In the supine position, the median [IQR] leak fractions for the i-gel and ETT groups were 31.99 [14.54] % and 26.06 [9.62] % (*P* = 0.341). In addition, there was significantly difference in the peak and mean airway pressure in the supine position for the i-gel and ETT groups (peak: 11.44 [1.43]; 12.53 [1.68], *P* = 0.03, mean: 4.67 [0.71]; 4.95 [0.46], *P* = 0.049). Furthermore, except airway pressure in supine position, there were no statistically significant differences in both positions between the groups in leak volume, compliance, or peak and mean airway pressures (Table 2). However, resistance differed between the groups for both positions, with the i-gel group exhibiting significantly lower values than those of the ETT group (*p* < 0.001).

**Fig. 3:**
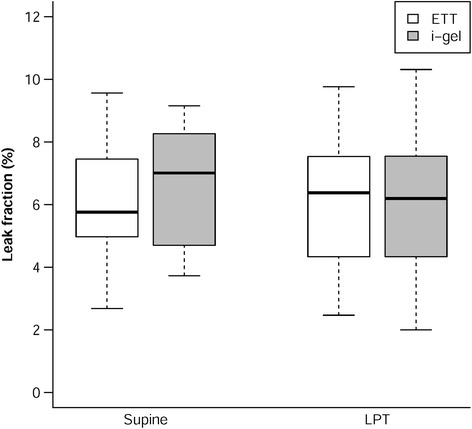
Boxplot of leak fraction for i-gel and ETT groups. Data are median [IQR]. ETT: endotracheal tube

For the i-gel group, less leakage (leak fraction: median [IQR]) was observed in the LPT position (6.20 [3.49] %) than in the supine position (7.01 [3.73] %) (*P* = 0.179), as illustrated in Fig. 3. For the ETT group, leakage (median [IQR]) was similar in both positions (supine : 5.76 [2.67] %; LPT: 6.38 [3.71]%, *P* = 0.194) (Table 2).

### Postoperative recovery follow-up

During surgery, none of the patients who received the i-gel were required to shift to intubation. In addition, vomitus was not noted in any patients following extubation or i-gel removal. In the postoperative care unit, the number of patients who reported a sore throat was significantly lower in the i-gel group (3) than in the ETT group (9; *p* = 0.038). No patients in either group reported any symptoms of aspiration or following respiratory complications, including cough, laryngospasm or reintubation in the postoperative care unit. They also did not report any of the aspiration following complications prior to discharge. All patients were discharged uneventfully.

## Discussion

Our findings indicate that the i-gel provides a suitable alternative to the ETT when using LPT positioning. The i-gel group produced similar leak fractions, leak volumes, and airway pressure to the ETT group. In addition, the i-gel group had significantly lower resistance and higher compliance compared with the ETT group. In addition, in the i-gel group, we observed lower leak fractions and volumes in the LPT position than in the supine position with our sample of non-obese patients. Following surgery we observed fewer cases of sore throat in the i-gel group than in the ETT group, and no clinical or endoscopic signs of aspiration in either groups.

In this study, we observed no clinical signs associated with aspiration in the i-gel group. This is because the i-gel has an esophageal channel, which enables the release of pressure induced by abdominal insufflation and head-down position during the perioperative period. In addition, the i-gel provides better sealing than first generation SADs. This means that the i-gel can completely separate the gastrointestinal and respiratory tracts and protect the airway safety, even if vomiting occurs [10, 13].

In the i-gel group, we observed a lower leak fraction and volume during laparoscopy when patients were in the Trendelenburg position compared with the supine position. Our finding is contrary to Carron et al. who found higher gas leakage using LMA-Proseal in laparoscopy with the reverse Trendelenburg position [25]. This suggests that the positioning change may be a major factor affecting gas leakage when using SADs.

**Fig. 4:**
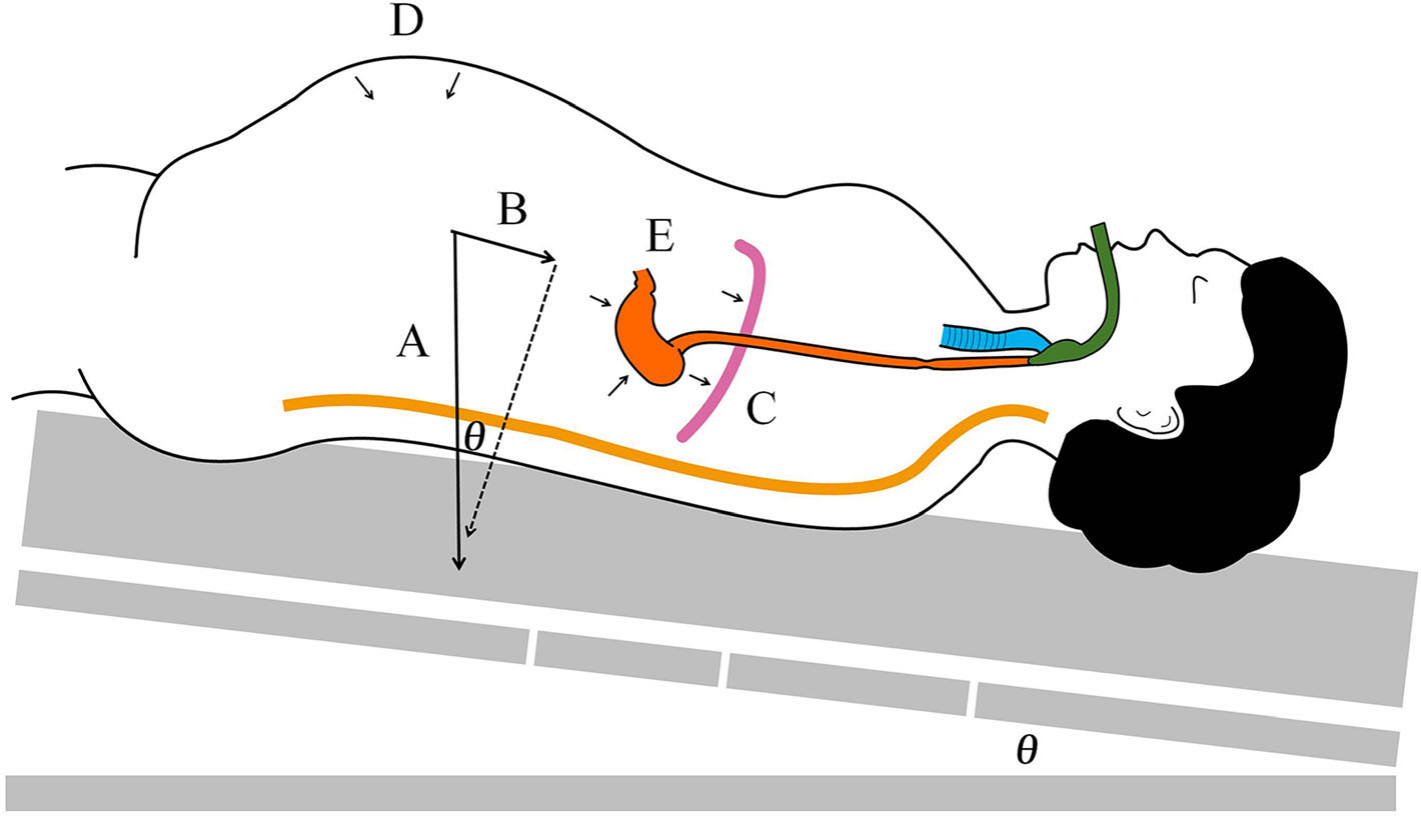
The force causes less leak fraction in the LPT position than in the supine position. *a* Body weight force. *b* Component of body weight and cephalic shifting pressure due to LPT position. *c* LPT position induced force on the diaphragm. *d* Force caused by pneumoperitoneum. *e* LPT position induced pressure on the stomach. LPT: Laparoscopic pneumoperitoneum and Trendelenburg

We propose two explanations for our findings. First, the partial body weight, cephalic viscera and diaphragm pressure caused by the pneumoperitoneum and Trendelenburg position (Force B as shown in Fig. 4) may result in a tighter seal in the LPT position compared with the supine position. Alternatively, it is possible that the airway undergoes a configuration change in the LPT position, yielding a superior sealing pressure with the i-gel [26].

In the LPT and supine positions, resistance was significantly lower in the i-gel group than in the ETT group. We propose that this result was caused by the internal diameter being larger than that of the ETT. The size four i-gel is shorter (192 mm) and has a larger cross section (internal diameter: 12.3 mm) than the size 7.0 ETT (length: 310 mm; internal diameter: 7.0 mm) [27]. According to the Poiseuille equation (resistance = (8 × length × viscosity) ∕ (π × radius^4^)) [28], the resistance of ETT is 15.34 times greater than the i-gel. In addition, in the supine position, airway pressure in ETT was significantly higher than i-gel group. It because the diameter of i-gel is larger than ETT due to Poiseuille equation, too.

In the postoperative care unit, we observed significantly lower rates of sore throat in the i-gel group than in the ETT group. This result is similar to the findings of previous research, which compared ETTs with other disposable SADs [29]. The non-inflatable cuff of the i-gel provides low mucosal pressure [30], and can prevent hyperinflation caused by the diffusion of gases, such as nitrous oxide [31]. This results in a decreased compression of the microvascular structures and terminal nerve endings in the peri-laryngeal tissues [32].

This study has some identifiable limitations. Although we did not observe any symptoms or signs of aspiration, a larger sample size is needed to confirm the safety of the i-gel. The incidence of aspiration with the LMA Classic has been estimated at 0.02%. This rate is similar to that obtained with tracheal intubation in elective patients [33]. Further investigation is also needed in patients with morbid obesity, symptoms of gastroesophageal reflux, and those undergoing operations of a longer duration. In addition, in the i-gel group, we found that the less leakage in the LPT position was noted than in the supine position, and this phenomenon is needed further investigated in the future.

## Conclusions

Our data supports that the i-gel provides a suitable alternative to the ETT for use in surgeries that use the LPT position. The i-gel provides a similar leak fraction and significantly lower resistance than the ETT. Moreover, the i-gel group displayed no evidence of aspiration, and reported lower incidences of sore throat.

## Additional files

Additional file 1: “Demographic and postoperative complication changes” This file contains raw data about demographic and postoperative complications. (XLSX 13 kb)
Additional file 2: “Respiratory parameters” This file contains raw data about the respiratory parameters, including leak fraction, leak volume, etc. (XLSX 14 kb)

## Abbreviations

ASA: American Society of Anesthesiologists
BMI: Body mass index
ETT: Endotracheal tube
LPT: Laparoscopic pneumoperitoneum and Trendelenburg
